# Local antibiotic carriers in the surgical management of pyogenic spondylodiscitis: First comparative evaluation of gentamicin-loaded versus vancomycin-loaded efficacy

**DOI:** 10.1007/s00132-025-04657-0

**Published:** 2025-05-13

**Authors:** Yu Xiao, Vincent Heck, Long Hao, Michael Rauschmann, Andrei Slavici

**Affiliations:** 1 Center for Spinal Surgery, Sana Klinikum Offenbach, Starkenburgring 66, 63069 Offenbach, Germany; 2 Department of Spinal Surgery, Fourth People’s Hospital of Guiyang, Guiyang, China; 3 Faculty of Medicine and University Hospital Cologne, Department of Orthopedic, Trauma and Plastic Surgery, University of Cologne, Kerpener Str. 62, 50937 Cologne, Germany

**Keywords:** Pyogenic Spondylodiscitis, Interbody Fusion, Local Antibiotic Therapy, Gentamicin, Vancomycin, Pyogene Spondylodiszitis, Interkorporelle Fusion, Lokale Antibiotikatherapie, Gentamicin, Vancomycin

## Abstract

**Objective:**

To evaluate the efficacy of intraoperative gentamicin versus vancomycin-loaded PerOssal (Osartis, Münster, Germany) carriers on interbody fusion rates and infection control in patients undergoing surgery for pyogenic spondylodiscitis.

**Methods:**

This retrospective study included 29 patients with pyogenic spondylodiscitis who underwent surgical debridement, interbody fusion, and pedicle screw fixation between February 2018 and March 2023. Patients received PerOssal carriers loaded with either gentamicin (Group A, *n* = 14) or vancomycin (Group B, *n* = 15). Clinical outcomes, including fusion rates, infection control, complications, and inflammatory markers, were analyzed.

**Results:**

Baseline characteristics between groups were comparable. Fusion rates at 3–6 months’ follow-up were 92.8% (13/14) in Group A and 80.0% (12/15) in Group B, without significant differences (*P* > 0.05). Both groups showed significant reductions in white blood cell counts and C‑reactive protein levels postoperatively, without inter-group differences (*P* > 0.05). Complications included cerebrospinal fluid leakage, hematoma, pulmonary embolism, and wound infections, all managed successfully with no recurrent infections observed.

**Conclusion:**

In the short term, PerOssal carriers loaded with either gentamicin or vancomycin demonstrated effective infection control for pyogenic spondylodiscitis and high interbody fusion rates. Moreover, no apparent adverse effects on bone healing were associated with the local administration of high-concentration antibiotics.

**Graphic abstract:**

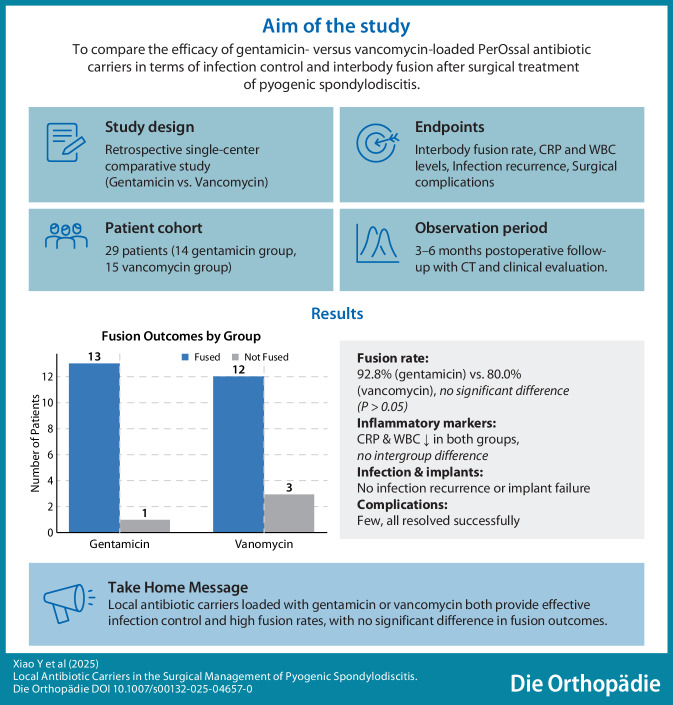

## Abstract (de)

**Zielsetzung:**

Zielsetzung dieser Studie war die Untersuchung der Wirksamkeit von intraoperativ eingesetzten, Gentamicin- bzw. Vancomycin-beladenen PerOssal-Trägern (Osartis, Münster, Deutschland) im Sinne der lokalen Antibiotikatherapie auf die interkorporelle Fusionsrate und Infektionskontrolle bei Patienten, die aufgrund einer pyogenen Spondylodiszitis operiert wurden.

**Methoden:**

Diese retrospektive Studie umfasste 29 Patienten mit pyogener Spondylodiszitis, die zwischen Februar 2018 und März 2023 einer chirurgischen Débridement, interkorporellen Fusion und Pedikelschraubenfixation unterzogen wurden. Die Patienten erhielten entweder mit Gentamicin (Gruppe A, *n* = 14) oder Vancomycin (Gruppe B, *n* = 15) beladene PerOssal-Träger. Klinische Ergebnisse wie Fusionsraten, Infektionskontrolle, Komplikationen und Entzündungsparameter wurden analysiert.

**Ergebnisse:**

Die Ausgangsmerkmale waren zwischen den Gruppen vergleichbar. Die Fusionsraten nach 3–6 Monaten Nachbeobachtung betrugen 92,8 % (13/14) in Gruppe A und 80,0 % (12/15) in Gruppe B, ohne signifikante Unterschiede (*p* > 0,05). Beide Gruppen zeigten postoperativ eine signifikante Reduktion der weißen Blutkörperchenzahl und des C‑reaktiven Proteins, ohne Unterschiede zwischen den Gruppen (*p* > 0,05). Komplikationen umfassten Liquorleckagen, Hämatome, Lungenembolien und Wundinfektionen, die alle erfolgreich behandelt wurden, ohne dass Rezidivinfektionen beobachtet wurden.

**Schlussfolgerung:**

Kurzfristig zeigten sowohl mit Gentamicin als auch mit Vancomycin beladene PerOssal-Träger eine effektive Infektionskontrolle bei pyogener Spondylodiszitis und hohe interkorporelle Fusionsraten. Zudem traten keine nachweisbaren negativen Auswirkungen der lokal applizierten hochdosierten Antibiotika auf die Knochenheilung auf.

## Introduction

Pyogenic spondylodiscitis refers to a purulent infection of the intervertebral disc and adjacent vertebral bodies caused by nonspecific bacterial pathogens. With the aging population, the increase in diabetes mellitus, and the use of immunosuppressive agents and steroids, the incidence has been steadily increasing [1, 2]. The reported incidence is 14.4 cases per 100,000 individuals, with 59.6% of cases occurring in patients aged 70 years or older; the lumbar spine is most commonly involved (56.2%) [3]. Clinical presentation typically includes nonspecific low back pain, sometimes accompanied by fever and neurological deficits, but these symptoms are not pathognomonic [4]. Consequently, a delay of several weeks or even months often occurs between the onset of symptoms and the time of diagnosis [5, 6].

In approximately 90% of cases, conservative treatment effectively eliminates the infection, relieves pain, and maintains spinal stability, preventing neurological dysfunction [7]. However, in patients with spinal instability whose infections are not controlled through conservative treatment, surgical intervention is widely considered a valid indication [8]. The main goals of surgery are thorough debridement of the intervertebral disc-space and management of abscess formation in the paravertebral region, to eradicate infection and subsequent interbody fusion with insertion of stabilizing implants, followed by pedicle screw fixation to restore spinal stability. Intraoperatively, antibiotic carriers loaded with gentamicin or vancomycin are frequently placed into the intervertebral space after adequate debridement. This technique has gained widespread clinical acceptance and has shown satisfactory outcomes [9, 10]. At high concentrations, both gentamicin and vancomycin have shown certain negative effects on osteoblasts in in vitro cell experiments, although vancomycin appears to be relatively safer, with minimal impact on osteoblasts at clinical doses [11]. Animal studies have demonstrated that vancomycin does not significantly affect spinal fusion outcomes, whereas gentamicin, particularly at higher doses, has been found to impair bone healing and fusion. Notably, delayed healing was observed in bone grafts loaded with gentamicin [12, 13]. To date, no study has addressed whether there are any differences in fusion rates when using the same type of carrier loaded separately with gentamicin or vancomycin.

Therefore, this study aims to compare the interbody fusion outcomes of antibiotic carriers, loaded with gentamicin versus vancomycin. The study results will further validate the effectiveness of locally applied antibiotic carriers loaded with different antibiotics. With the availability of different antibiotics as local treatments and an understanding of the bacteria responsible for spondylodiscitis, a more targeted therapy against the specific pathogens can be achieved.

## Methods

This study was designed as a single-center, retrospective investigation. Clinical data were reviewed for patients who underwent debridement and interbody fusion with internal fixation for pyogenic spondylodiscitis at the authors’ hospital between February 2018 and March 2023. During surgery, a synthetic antibiotic carrier (PerOssal, Osartis, Münster, Germany) loaded with either gentamicin or vancomycin was applied.

Based on the type of antibiotic used in the PerOssal carrier, patients were stratified into two groups: the gentamicin group (Group A) and the vancomycin group (Group B).

### Inclusion criteria

- Clinical symptoms before surgery.
- Elevated white blood cell (WBC) count and C‑reactive protein (CRP) levels on laboratory testing.
- Magnetic resonance imaging (including contrast-enhanced T1-weighted sequences) and computed tomography (CT) scans confirming infection.
- Positive pathogen cultures (e.g., blood culture prior to surgery, percutaneous biopsy, or surgical swabs for microbiological testing) or positive pathological results obtained during surgery, confirming a diagnosis of pyogenic spondylitis.

### Exclusion criteria

- Patients who underwent only spinal canal decompression and debridement or did not receive interbody fusion.
- Patients with infections caused by *Mycobacterium tuberculosis, Brucella*, or fungal pathogens.

### Surgical procedure

All surgeries were performed by four experienced spine surgeons at our center. The surgical approach, fusion, and fixation segments were determined based on the location of the spinal lesion, the extent of vertebral destruction, and the presence of abscess formation.

- *Anterior approach:* For the cervical spine, an anterior cervical corpectomy and fusion (ACCF) was performed with anterior plating.
- *Posterior approach:* Involves debridement through the spinal canal and/or the intervertebral foramen, followed by intervertebral fixation with cages, filled with bone substitutes and posterior pedicle screw fixation.
- *Combined approaches:* For lumbar interbody fusion (ALIF), thoracolumbar interbody fusion (ATIF), or extreme lateral interbody fusion (XLIF), the anterior approach was combined with posterior open or percutaneous pedicle screw fixation.

Once the intervertebral lesion was exposed, multiple tissue samples were collected for histological and microbiological testing. Thorough debridement was performed by removing most of the granulation tissue from the intervertebral disc space and the cartilage of the endplates. After sufficient irrigation, PerOssal carrier particles loaded with either gentamicin or vancomycin (400 mg of vancomycin or 160 mg of gentamicin per carrier) were implanted into the intervertebral space and interbody fusion devices (titanium cage or mesh), along with a mixture of allogeneic bone graft particles.

The choice of antibiotic-loaded carrier was based on preoperative bacterial culture results or on the patient’s medical history to select the appropriate antibiotic. In cases where preoperative cultures were negative and no relevant history was available, empirical antibiotic selection was made. For osteoporotic patients, bone cement was used to reinforce the screws at the upper and lower vertebral bodies.

### Postoperative management

All patients received intravenous antibiotic therapy for 3–8 weeks, followed by 4–8 weeks of oral antibiotics. Specific antibiotic regimens were adjusted based on the results of bacterial culture and drug susceptibility testing. For culture-negative cases, broad-spectrum antibiotics were administered to cover both Gram-positive and Gram-negative organisms. Patients wore a brace for 3 months postoperatively to protect the surgical site and were encouraged to begin early functional exercises. Follow-up CT scans were performed 3–6 months after surgery to evaluate interbody fusion. Two senior spine surgeons independently assessed the imaging results and reached a final consensus on fusion status. This standardized postoperative management pathway ensured effective infection control and facilitated successful interbody fusion.

## Outcome measures

### Radiological evaluation

**Fig. 1:**
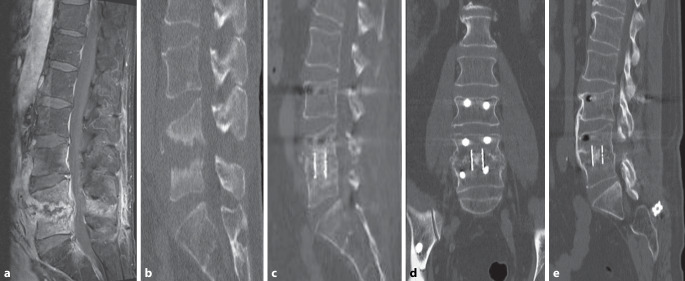
A 60-year-old patient diagnosed with lumbar pyogenic spondylodiscitis at L4–5 underwent posterior debridement and interbody fusion, with a gentamicin-loaded antibiotic carrier applied intraoperatively. **a**,**b** Preoperative MRI and CT scans demonstrate destruction of the L4–5 disc space and adjacent vertebral bodies. **c**,**d** At 3 months postoperatively, the patient showed Bridwell grade 3 fusion. **e** At 16 months postoperatively, CT scans reveal complete bony fusion at the treated level

**Fig. 2:**
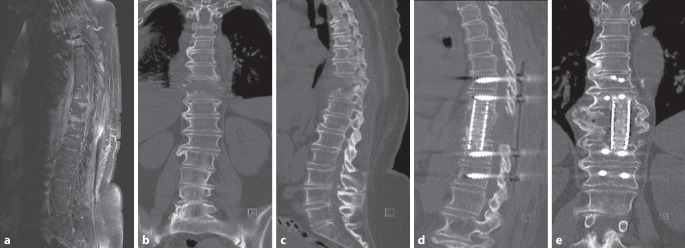
A 69-year-old patient with pyogenic spondylodiscitis at T9–10 underwent posterior debridement and interbody fusion, with a vancomycin-loaded antibiotic carrier applied intraoperatively. **a–c** Preoperative MRI and CT scans show destruction of the T9–10 intervertebral disc and adjacent vertebral bodies. **d**,**e** At 3 months postoperatively, the patient achieved Bridwell grade 2 fusion. The cage remained well positioned, and notable new bone formation within the fusion site suggested the onset of osseous healing

Interbody fusion was assessed by CT scans at a single follow-up visit occurring between 3 and 6 months postoperatively. The assessment followed the criteria proposed by Bridwell et al. [14], which focus on bony bridging between adjacent vertebral bodies or new bone formation within the intervertebral space. According to this system, Grade I indicates solid fusion with remodeling and trabeculae present; Grade II indicates an intact graft not fully remodeled but without lucency; Grade III indicates a graft with definite lucency; and Grade IV indicates no fusion with graft resorption or collapse. (In response to the reviewer’s comment, we have added a brief explanation of the Bridwell classification system in the Radiological Evaluation section to clarify the grading criteria.) Fusion was defined as Bridwell grades 1 and 2 on CT scans, whereas grades 3 and 4 were considered nonfusion (Figs. 1 and 2). The CT images were analyzed by two senior spine surgeons, and final fusion status was determined through consensus.

### Clinical and laboratory outcomes

Clinical follow-up included evaluating pain relief, wound healing, and local pain at the surgical site. At discharge, white blood cell (WBC) count and C‑reactive protein (CRP) levels were recorded to assess infection control.

### Statistical analysis

All statistical analyses were performed using SPSS software (version 29.0; IBM Corp., Armonk, NY, USA). The normality of continuous variables was assessed using the Shapiro–Wilk test. Continuous variables are expressed as the mean ± standard deviation (mean ± SD), whereas categorical variables are presented as counts (percentages). For between-group comparisons, an independent-samples t‑test was used for normally distributed data, and the Mann–Whitney U test was applied for data that were not normally distributed. Categorical variables were compared using either the chi-square (χ^2^) test or Fisher’s exact test.

To evaluate the robustness of the primary outcome measures (e.g., interbody fusion rate), sensitivity analyses were performed by excluding older patients (> 80 years) and those with severe comorbid conditions (e.g., diabetes mellitus or chronic renal failure) were analyzed using the kappa (κ) statistic; a κ value greater than 0.75 was considered to indicate excellent agreement. For all statistical analyses, a *p*-value < 0.05 was regarded as statistically significant.

## Results

### Baseline clinical presentation

Most patients presented with axial back pain (89.6%) at their initial visit. All patients had single-segment lesions, primarily affecting the lumbar spine (58.62%), followed by the thoracic spine (17.24%), thoracolumbar spine (17.24%), and cervical spine (14.7%). Additional complications included psoas abscess (20.7%), epidural abscess (13.8%), paraspinal abscess (6.9%), and iliac fossa abscess (3.4%). Frequent comorbidities included diabetes mellitus (34.5%), obesity (20.7%), chronic renal failure (13.8%), chronic glucocorticoid use (3.4%), chronic immunosuppressive status (3.4%), and cirrhosis (3.4%). A history of long-term smoking was noted in 41.38% of patients. At the time of admission, elevated CRP levels were found in 93.1% of patients, and elevated WBC counts were observed in 41.38% of cases.

### Interbody fusion and infection control

**Fig. 3:**
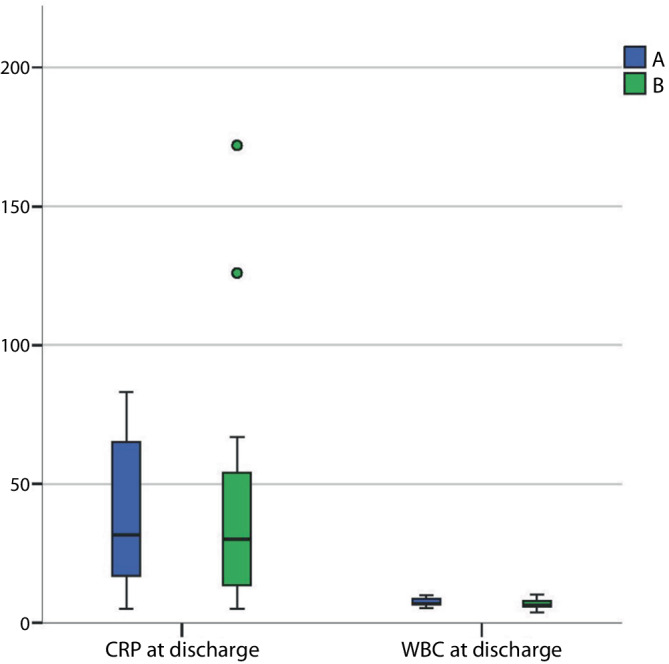
Distribution of C‑reactive protein (CRP) at discharge and white blood cell count (WBC) at discharge for groups A and B

No statistically significant differences were found in baseline demographics (e.g., age, sex, body mass index [BMI], smoking status, Charlson Comorbidity Index) or infection sites between Group A and Group B (*P* > 0.05; Table 1). Additionally, there were no significant differences in preoperative WBC and CRP levels (*P* > 0.05; Table 1). During follow-up, the fusion rate in Group A was 92.8% (13/14), which included 3 patients achieving complete fusion and 10 exhibiting partial fusion, leaving 1 segment unfused. The fusion rate in Group B was 80.0% (12/15), consisting of 2 patients with complete fusion, 10 with partial fusion, and 3 remaining unfused. No significant difference in fusion rates was found between the two groups (*P* > 0.05; Table 1). By the time of discharge, both WBC and CRP levels had decreased significantly from admission (*P* < 0.05), and no statistically significant differences were observed between the groups (*P* > 0.05; Table 2; Fig. 3). No implant failures (e.g., implant fracture) occurred during follow-up, and there were no clinical signs of recurrent infection, such as persistent pain or wound erythema. Bone cement augmentation of pedicle screws was performed in 2 osteoporotic patients (6.9%).

**Table 1:** Basic characteristics and preoperative data of group A and group B

	A	B	Between-group difference (95%CI)	*P*
*Age*	72.71 ± 9.90	71.53 ± 7.46	1.18 (−5.47 to 7.83)	0.948
*BMI*	26.02 ± 3.71	28.58 ± 5.14	−2.56 (−5.81 to 0.69)	0.169
18.5 < BMI < 25, *N* (%)	7 (50)	6 (40)		
25 < BMI < 30, *N* (%)	5 (35.7)	3 (20)		
30 < BMI < 35, *N* (%)	2 (14.3)	6 (40)		
*Gender, N (%)*				
Women	4 (28.6)	6 (40)	–	0.7
Men	10 (71.4)	9 (60)	–	
*Affected levels, N (%)*				
Cervical spine	1 (7.1)	0	–	0.558
Thoracic spine	4 (28.8)	4 (26.7)	–	
Lumbosacral spine	9 (64.3)	11 (73.3)	–	
*Smoking habit, N (%)*				
Yes	8 (57.1)	4 (26.7)	–	0.139
No	6 (42.9)	11 (73.3)	–	
*CCI, N (%)*				
1	1 (7.1)	0	–	0.359
2	1 (7.1)	1 (6.7)	–	
3	1 (7.1)	1 (6.7)	–	
4	4 (28.6)	1 (6.7)	–	
5	0	3 (20)	–	
6	2 (14.3)	3 (20)	–	
7	1 (7.1)	3 (20)	–	
8	3 (21.4)	1 (6.7)	–	
9	0	1 (6.7)	–	
10	0	1 (6.7)	–	
11	1 (7.1)	0	–	
*Follow-up (months)*	3.71 ± 1.27	3.53 ± 1.06	0.18 (−0.67 to 1.03)	0.88
*Bridwell grade, N (%)*				
I	2 (21.4)	2 (13.3)	–	0.558
II	10 (71.4)	10 (66.7)	–	
III	1 (7.1)	3 (20)	–	
*CRP admission*	83.43 ± 101.53	140.6 ± 107.23	−57.17 (−133.15 to 18.81)	0.058
*WBC admission*	9.69 ± 2.80	9.61 ± 3.56	0.08 (−2.37 to 2.53)	0.718

Data are given as mean ± standard deviation or number (*N*)

*CI* confidence interval, *CCl* Charlson comorbidity index, *BMI* Body Mass Index, *CRP* C‑reactive protein, *WBC* white blood cell count

**Table 2:** C‑reactive protein (CRP) and white blood cell count (WBC) at discharge of group A and group B

	A	B	Between-group difference (95%CI)	*P*
CRP at discharge	43 ± 27.64	45.01 ± 46.86	−2.01 (−29.80 to 25.78)	0.896
WBC at discharge	7.38 ± 1.38	6.67 ± 1.73	0.71 (−0.49 to 1.91)	0.233

Data are given as mean ± standard deviation or number

*CI* confidence interval, *CRP* C‑reactive protein, *WBC* white blood cell count

### Pathogen distribution

Among 28 patients who underwent intraoperative specimen collection for bacterial culture, 17 yielded positive results. Of the 12 patients who underwent blood culture, 6 tested positive. Five patients underwent CT-guided biopsy for bacterial culture, resulting in 2 positive findings. Collectively, these three methods identified bacterial pathogens in 21 patients (72.4%). One patient exhibited multiple strains of Gram-positive bacteria. *Staphylococcus aureus* was the most frequently isolated pathogen. A detailed breakdown of the isolated organisms is provided in Table 3.

**Table 3:** Microbiologic findings

Bacteria	Cases
Gram-positive bacteria	
*Staphylococcus aureus*	4
*Enterococcus faecalis*	2
*MRSE*	2
*Propionibacterium acnes (Cutibacterium acnes)*	2
*Streptococcus anginosus*	2
*Actinomyces neuii*	1
*Staphylococcus lugdunensis*	1
*Streptococcus constellatus*	1
*Staphylococcus hominis MR*	1
*Staphylococcus epidermidis*	1
*Streptococcus intermedius*	1
Gram-negative bacteria	
*Enterobacter cloacae*	1
*Escherichia coli*	1
*Klebsiella aerogenes*	1

*MRSE* Methicillin-resistant *Staphylococcus epidermidis*

### Intraoperative and postoperative complications

One patient experienced an intraoperative cerebrospinal fluid leak, which was repaired during the procedure. Another patient developed a compressive hematoma in the lumbar region during the early postoperative period and underwent successful hematoma evacuation. One patient experienced a pulmonary embolism, with symptoms improving following appropriate treatment. Two patients developed postoperative wound infections and underwent debridement and irrigation; both subsequently achieved satisfactory wound healing. Three patients presented with wound edge necrosis, which was managed by local debridement under anesthesia; all wounds ultimately healed well.

## Discussion

The findings of this study indicate that both Group A and Group B achieved high fusion rates during follow-up, with no signs of recurrent infection. These results are consistent with previous literature, which similarly reported satisfactory antibiotic–carrier absorption and bone fusion 3–6 months postoperatively in patients with spondylodiscitis [15]. Consequently, it appears that in a comprehensive therapeutic setting—encompassing thorough debridement, intervertebral stabilization, stable internal fixation, and individualized antibiotic selection—the local application of high-concentration antibiotics does not significantly inhibit interbody fusion and or wound healing.

Controversies regarding the potential inhibition of bone healing by locally administered, high-concentration antibiotics primarily arise from animal models and in vitro studies. Findings from these models suggest that the rapid release of gentamicin may interfere with bone healing [13], while higher concentrations of vancomycin can markedly reduce the viability of bone marrow-derived mesenchymal stem cells, inhibiting their osteogenic differentiation [16]. Additionally, in a study of contaminated posterior lateral spinal fusion, demineralized bone matrix loaded with vancomycin showed potent antimicrobial activity but slightly weaker fusion capability compared to demineralized bone matrix alone [17].

Despite these concerns, multiple clinical studies have reported satisfactory fusion rates, when high local antibiotic concentrations are used. Tang et al. [18] demonstrated that local application of vancomycin (1.0 g) mixed with gelatin, combined with conventional bone grafting methods, yielded successful fusion in all patients. Another study employed antibiotic-loaded calcium sulfate beads for spondylodiscitis, finding new bone formation and fusion 3–6 months postoperatively, with no recurrences of infection [19]. Similarly, when PerOssal materials containing antibiotics were used, bone fusion was achieved in all patients by 3–6 months, and no signs of infection were observed [15]. In line with these reports, our study revealed no significant differences between the two groups in short-term radiographic fusion rates.

It should be noted that interbody fusion is a complex biological process, with local high-concentration antibiotics representing only one of many influencing factors. Thorough debridement, for instance, removes pathogenic microbes and inflammatory mediators, creating a cleaner and more stable environment for bone growth. The mechanical stability provided by pedicle screw fixation further enhances local blood supply, facilitating the delivery of osteogenic cells and growth factors. Moreover, gentamicin concentrations as high as 400 mg/mL [20] and vancomycin concentrations below 1000 µg/mL have been shown to exhibit minimal negative effects on the metabolic activity of human osteoblasts [21]. Over time, antibiotic levels gradually decline, reducing any potential interference with bone fusion.

The PerOssal material used in this study consists mainly of nano-hydroxyapatite and calcium sulfate (mass ratio 60:40). Nano-hydroxyapatite provides osteoconductivity, promoting bone regeneration [22]; PerOssal acts as an absorbable antibiotic carrier with superior antibiotic release kinetics compared to pure calcium sulfate alone, exhibiting a gentamicin release rate as high as 94.7% within 10 days [23]. Compared with traditional polymethylmethacrylate (PMMA) bone cement, PerOssal is gradually absorbed by the body, avoiding the disadvantage of PMMA, which typically requires a second surgery for removal. Additionally, it prevents PMMA’s drawback of potentially becoming a bacterial attachment site when antibiotic concentrations fall below the minimum inhibitory concentration. PerOssal has been successfully used in the surgical treatment of osteomyelitis in long bones [24] and spondylodiscitis [15]. A prospective study further demonstrated that the intraoperative application of PerOssal antibiotic carriers can effectively reduce the dose and duration of systemic antibiotic therapy, thus, minimizing drug-related hepatotoxicity and nephrotoxicity [25]. Our study observed satisfactory clinical outcomes while confirming that the local administration of two different antibiotics did not result in significant differences in interbody fusion outcomes.

In this study, 72.4% of the patients yielded positive bacterial cultures through a combination of intraoperative specimens, blood cultures, and CT-guided biopsy. Gram-positive germs were most frequently isolated, although a few Gram-negative organisms were detected. Consistent with previous reports, *Staphylococcus aureus* remained the most common pathogen in pyogenic spondylodiscitis [26]. Several resistant strains, such as methicillin-resistant *Staphylococcus epidermidis* (MRSE) and methicillin-resistant *Staphylococcus hominis*, were also identified, emphasizing the need for vigilance regarding drug-resistant pathogens in clinical practice. Although Gram-positive bacteria account for the majority of cases in most studies, Gram-negative organisms are not uncommon in immunocompromised patients or those with chronic renal failure or diabetes [27]. In this study, for cases with negative cultures or suspected drug-resistant organisms, a strategy combining intraoperative local antibiotic application with postoperative intravenous antibiotic therapy was adopted, to provide comprehensive coverage against potential Gram-positive and Gram-negative pathogens during the perioperative period. Commonly used intravenous antibiotic regimens include broad-spectrum antibiotics (such as vancomycin, combined with rifampin, or clindamycin, combined with rifampin), or a combination of antibiotics (such as piperacillin/tazobactam). No recurrence of infection was observed during the follow-up period, indicating the effectiveness and safety of this approach in clinical practice.

Concerning the fusion rates, it can be considered that although fusion was not complete even after 3–6 months, this does not mean that pseudarthrosis will occur. Due to creeping substitution processes, in the following 12 months after surgery, the increase of fusion rates can be seen [28, 29]. Since we do not routinely perform follow-up CT scans at 12 months in asymptomatic patients at our institution, data at that timepoint are not available.

## Limitations and future directions

This study primarily evaluated the interbody fusion rate and clinical recurrence of infection over a short follow-up period (3–6 months). Due to the relatively brief observation window, the long-term stability of the fixation system and recurrence of infection could not be fully assessed. Moreover, the limited sample size may affect the generalizability of the findings. Future research should involve multicenter, large-scale, prospective trials with extended follow-up that includes additional inflammatory markers and imaging analyses. Such studies would more comprehensively determine the mid- to long-term safety and efficacy of local antibiotic carriers in facilitating bone fusion and preventing infection. Furthermore, the antibiotic concentration in the wound fluid should be measured and compared to the blood concentration of the antibiotic substance. Such studies would more comprehensively determine the safety and efficacy of local antibiotic carriers in facilitating bone fusion and preventing infection.

## Conclusion

Treating pyogenic spondylodiscitis with PerOssal carriers loaded with gentamicin or vancomycin resulted in a high interbody fusion rate and effective short-term infection control. No significant differences in fusion rates or perioperative infection control were observed between the two antibiotics, and no clear adverse effects of locally administered high-concentration antibiotics on bone healing were detected. This study provides a reliable basis for the individualized use of different types of antibiotic carriers. It further suggests that, when combined with adequate debridement, stable internal fixation, and carefully selected antibiotic regimens, the local application of antibiotic carriers is both safe and effective. In the future, this treatment concept can lead to the opportunity to reduce the length of systemic antibiotic therapy, due to the effectiveness of the local antibiotic therapy, which needs to be proven by further prospective randomized studies.

## Data Availability

The datasets generated and/or analyzed during the current study are available from the corresponding author upon reasonable request.

## Abbreviations

ACCF: Anterior Cervical Corpectomy and Fusion
ALIF: Anterior Lumbar Interbody Fusion
ATIF: Anterior Thoracolumbar Interbody Fusion
BMI: Body Mass Index
CRP: C-Reactive Protein
CT: Computed Tomography
MRSE: Methicillin-Resistant Staphylococcus epidermidis
MRI: Magnetic Resonance Imaging
PMMA: Polymethylmethacrylate
SD: Standard Deviation
WBC: White Blood Cell count
XLIF: Extreme Lateral Interbody Fusion

## Declarations

This study was approved by the relevant institutional ethics committee (approval no.: 2022-2902-evBO).
